# Identification of metabolites with anticancer properties by computational metabolomics

**DOI:** 10.1186/1476-4598-7-57

**Published:** 2008-06-17

**Authors:** Adrian K Arakaki, Roman Mezencev, Nathan J Bowen, Ying Huang, John F McDonald, Jeffrey Skolnick

**Affiliations:** 1 Center for the Study of Systems Biology, Georgia Institute of Technology, Atlanta, Georgia, USA; 2 School of Biology, Georgia Institute of Technology, Atlanta, Georgia, USA; 3 Ovarian Cancer Institute, Georgia Institute of Technology, Atlanta, Georgia, USA

## Abstract

**Background:**

Certain endogenous metabolites can influence the rate of cancer cell growth. For example, diacylglycerol, ceramides and sphingosine, NAD^+ ^and arginine exert this effect by acting as signaling molecules, while carrying out other important cellular functions. Metabolites can also be involved in the control of cell proliferation by directly regulating gene expression in ways that are signaling pathway-independent, e.g. by direct activation of transcription factors or by inducing epigenetic processes. The fact that metabolites can affect the cancer process on so many levels suggests that the change in concentration of some metabolites that occurs in cancer cells could have an active role in the progress of the disease.

**Results:**

CoMet, a fully automated Computational Metabolomics method to predict changes in metabolite levels in cancer cells compared to normal references has been developed and applied to Jurkat T leukemia cells with the goal of testing the following hypothesis: *Up or down regulation in cancer cells of the expression of genes encoding for metabolic enzymes leads to changes in intracellular metabolite concentrations that contribute to disease progression*. All nine metabolites predicted to be lowered in Jurkat cells with respect to lymphoblasts that were examined (riboflavin, tryptamine, 3-sulfino-L-alanine, menaquinone, dehydroepiandrosterone, α-hydroxystearic acid, hydroxyacetone, seleno-L-methionine and 5,6-dimethylbenzimidazole), exhibited antiproliferative activity that has not been reported before, while only two (bilirubin and androsterone) of the eleven tested metabolites predicted to be increased or unchanged in Jurkat cells displayed significant antiproliferative activity.

**Conclusion:**

These results: a) demonstrate that CoMet is a valuable method to identify potential compounds for experimental validation, b) indicate that cancer cell metabolism may be regulated to reduce the intracellular concentration of certain antiproliferative metabolites, leading to uninhibited cellular growth and c) suggest that many other endogenous metabolites with important roles in carcinogenesis are awaiting discovery.

## Background

Elucidating the metabolic changes exhibited by cancer cells is important not only for diagnostic purposes, but also to more deeply understand the molecular basis of carcinogenesis, which could lead to novel therapeutic approaches. By regulating the expression of oncogenes or modulating various signal transduction systems, it is widely accepted that certain metabolic processes play fundamental roles in cancer progression. The significance of other metabolic phenotypes observed in cancer is more controversial, e.g. the shift in energy production from oxidative phosphorylation (respiration) to aerobic glycolysis known as the Warburg effect [1]. The mainstream view has been that the Warburg effect is a consequence of the cancer process (secondary events due to hypoxic tumor conditions) rather than a mechanistic determinant, as originally hypothesized. Recently, however, a different picture of the role of metabolic changes in tumorigenesis has emerged; for example, the dichloroacetate-induced reversion from a cytoplasm-based glycolysis to a mitochondria-located glucose oxidation inhibits cancer growth, supporting the idea that the glycolytic shift is a fundamental requirement for cancer progression [2] and opening up the possibility of targeting metabolic pathways for cancer treatment [3].

Changes in intracellular concentrations of certain metabolites can influence the rate of cancer cell growth. A metabolite can exert this effect by acting as a signaling molecule, a role that does not preclude other important cellular functions. For instance, diacylglycerol, a lipid that confers specific structural and dynamic properties to biological membranes and serves as a building block for more complex lipids is also an essential second messenger in mammalian cells whose dysregulation contributes to cancer progression [4]. Similarly, structural components of cell membranes such as ceramides and sphingosine are also second messengers with antagonizing roles in cell proliferation and apoptosis [5]. Pyridine nucleotides constitute yet another example, having well characterized functions as electron carriers in metabolic redox reactions and roles in signaling pathways [6]. In particular, NAD^+ ^modulates the activity of sirtuins, a recently discovered family of histone deacetylases [7] that may contribute to breast cancer tumorigenesis [8]. Arginine is yet another metabolite involved in numerous biosynthetic pathways that also has a fundamental role in tumor development, apoptosis and angiogenesis [9]. Considering that the signaling role of many of these biomolecules was not even suspected a decade ago, it is likely that the role of other metabolites as second messengers will be discovered.

It is becoming increasingly clear that cellular metabolites can also be involved in the control of cell proliferation by directly regulating gene expression. Signaling pathway-independent modulation of gene expression by metabolites can occur on three levels [10]: First, metabolites can bind to regulatory regions of certain mRNAs (riboswitches), inducing allosteric changes that regulate the transcription or translation of the RNA transcript; however, this type of direct metabolite-RNA interaction has not yet been detected in humans [11]. Second, transcription factors can be activated upon metabolite binding, e.g. binding of steroid hormones to the estrogen receptor transcription factor induces gene expression events leading to breast cancer progression [12]. Third, metabolites can be involved in epigenetic processes such as post-translational modification of histones that regulate gene expression by changing chromatin structure [13]. The modulation of the rate of histone acetylation by nuclear levels of acetyl-CoA is an example of metabolic control over chromatin structure that involves epigenetic changes linked to cell proliferation and carcinogenesis [14].

The fact that metabolites can affect the cancer process on so many levels suggests that the manipulation of specific metabolic pathways may offer a reasonable therapeutic approach. In fact, this is the basis of several anticancer therapies that: i) have been proposed based on experimental evidence, ii) are currently the object of validation in clinical trials, or iii) are presently in clinical use. The inactivation of the metabolic enzymes KIAA1363 [15] and indoleamine 2,3-dioxygenase [16] constitutes a good example of i). As for ii), several anticancer treatments that exploit the antiproliferative action of ceramide are examples of therapies based on the pharmacological manipulation of a metabolic pathway that are currently in clinical trials [5]. Turning to iii), a metabolite-based therapy for acute lymphoblastic leukemia used since 1970 [17] consists of depleting circulating asparagine by administration of the bacterial enzyme L-asparaginase.

The analysis of metabolic features associated with neuroendocrine cancers by a combination of experimental techniques (magic angle spinning NMR spectroscopy and microanalytical biochemical assays) and *in silico *methods (reconstruction of metabolic pathways from microarray gene expression data and predictions of possible biotransformations based on the chemical groups present in a given metabolite) have resulted in a promising metabolome-directed therapy [18]. The goal is the detection of unusual pathways in the reconstructed metabolism of the cancer cell whose components can be targeted by already available drugs. In general, preventive and therapeutic anticancer approaches based on the pharmacological manipulation of metabolism aim to increase or decrease the intracellular levels of certain metabolites by administration of either the metabolites themselves, inhibitors/activators of relevant enzymes, or inhibitors/activators of specific transporters.

In this study, we hypothesize that the change in concentration of some metabolites that occurs in cancer cells could have an active role in the progress of the disease rather than merely being an inconsequential side effect. We explore whether the reversion to a metabolic phenotype more similar to the normal state might be of possible therapeutic value. Increasing the levels of certain compounds that are lowered in cancer cells could be straightforwardly achieved by directly administering the deficient metabolite. On the other hand, for metabolites whose levels are increased in cancer cells, reversion would involve activation or inhibition of key enzymes, an approach that is more difficult to implement. For that reason, here we decided to focus on the former case. Ideally, we would like to compare the actual intracellular levels of every human metabolite in normal and diseased states to identify those that are lowered in cancer cells. However, direct large-scale biochemical assays are currently unfeasible. Metabolite profiling based on NMR [19] or mass spectrometry techniques [20], although very powerful, require costly instruments and are not free of problems and limitations. *In silico *methods based on linking enzymes to upregulated microarray-detected transcripts and mapping to metabolic pathways have been applied to the qualitative reconstruction of the metabolome of cancer cells and some predictions have been successfully validated by biochemical experiments [18]. Here, we describe CoMet, a fully automated and general Computational Metabolomics method that uses a Systems Biology approach to predict the human metabolites whose intracellular levels are more likely to be altered in cancer cells. We then prioritize the metabolites predicted to be lowered in cancer compared to normal cells as potential anticancer agents. We applied our methodology to a leukemia cell line and discovered several human metabolites that either alone or in combination, exhibit various degrees of antiproliferative activity.

## Results

### Computational Metabolomics of Jurkat T cell line

**Figure 1:**
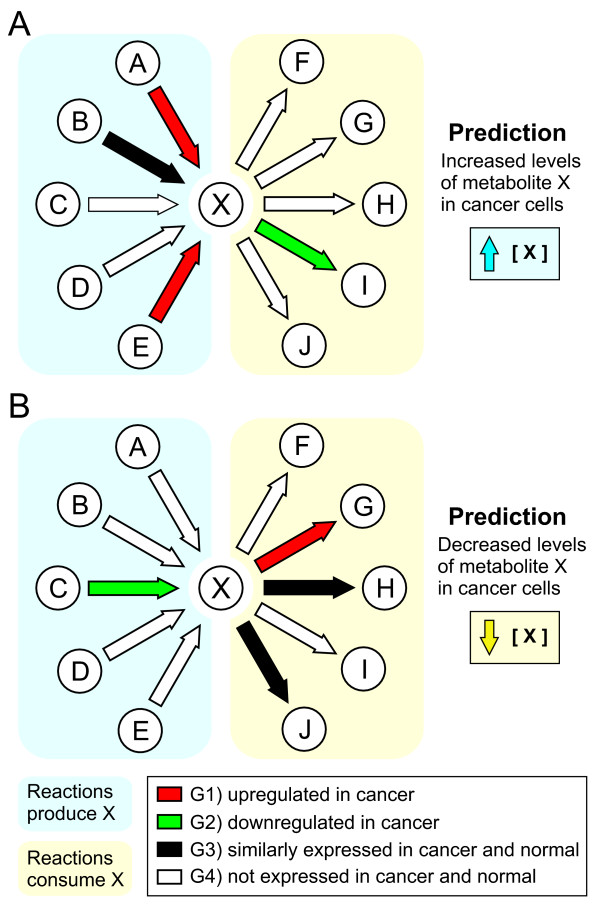
**Diagram representing the rationale of CoMet**. (A) The intracellular level of a metabolite X is predicted to be increased in cancer cells when enzymes that produce X are upregulated and/or enzymes that consume X are downregulated in cancer cells. (B) The intracellular level of a metabolite X is predicted to be decreased in cancer cells when enzymes that produce X are downregulated and/or enzymes that consume X are upregulated in cancer cells. See Material and Methods for a complete description of the rules.

The basic idea behind CoMet is schematically described in Figure 1. The intracellular level of a given metabolite is predicted to be decreased or increased in cancer cells based on the analysis of the relative expression levels of the human genes encoding for all identified enzymes that employ the metabolite as substrate or product (see Material and Methods for details). We applied our approach to the Jurkat cell line, which is derived from an acute T lymphoblastic leukemia patient [21]. The comparison of two Jurkat cell samples to three GM15851 lymphoblast cell samples (see Additional File 1) resulted in 104 metabolites predicted to be lowered in Jurkat cancer cells and 78 metabolites predicted to be increased in these cells, out of a total of 982 metabolites considered in the analysis (see Additional File 2). According to our hypothesis, we would expect an enrichment of Jurkat antileukemic agents among the 104 metabolites predicted to be lowered in the cancer cells. By performing an exhaustive search of the literature for experimental evidence, we found that 13 of the 982 analyzed metabolites has previously been demonstrated to exhibit anticancer activity in Jurkat cells. Table 1 shows that 2/13 metabolites are predicted to be lowered in Jurkat cells: thymidine, an antineoplastic agent [22], and prostaglandin D2, which induces apoptosis without inhibiting the viability of normal T lymphocytes [23]. Only 1/13 proven anticancer agents in Jurkat cells belongs to the group of 78 metabolites predicted to be increased in these cancer cells: the apoptotic agent 17β-2-methoxyestradiol [24]. The remaining 10 known anticancer molecules active in Jurkat cells: testosterone [25], melatonin [26], sphingolipid GD3 [27], 2'-deoxyguanosine [28], 2'-deoxyadenosine [29], 2'-deoxyinosine [29], nicotinamide [30], methylglyoxal [31], linoleic acid [32] and cAMP [33] are included in the set of 800 metabolites whose intracellular levels are predicted to be essentially the same in both Jurkat and normal cells. Although the fraction of metabolites with known anticancer activity among the compounds predicted to be lowered in Jurkat cells (2/104 = 0.019) is higher than that corresponding to the rest of the compounds [11 non predicted ones have literature validated anticancer properties; (1+10)/(78+800) = 0.013], the difference is not statistically significant (two-tailed Fisher's exact test at a critical alpha level of 0.05). On the other hand, it has to be noticed that negative results tend to be underreported, making it difficult to obtain unbiased statistics about metabolites that lack anticancer properties.

**Table 1:** Active metabolites predicted to be lowered in Jurkat cells

**Previously known anticancer activity in Jurkat cells**
thymidine (C00214)^1^
prostaglandin D2 (C00696)
**Anticancer activity in Jurkat cells tested in this work**

riboflavin (C00255)
tryptamine (C00398)
3-sulfino-L-alanine (C00606)
menaquinone (C00828)
dehydroepiandrosterone sulfate (C04555)
α-hydroxy fatty acid (C05102)
hydroxyacetone (C05235)
seleno-L-methionine (C05335)
α-ribazole (C05775)

^1 ^KEGG ligand identifier

### Tested metabolites predicted to be lowered in Jurkat cells are antiproliferative

**Figure 2:**
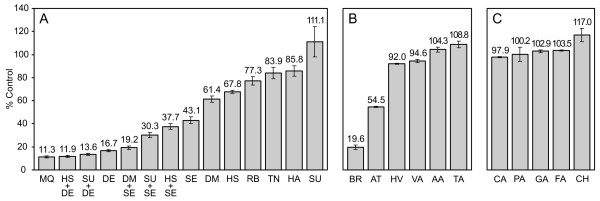
**Effect of endogenous metabolites on the proliferation of Jurkat cells**. The percentage of surviving cells is given as a percentage of the number of control cells after 72 h of incubation in the presence of the tested metabolite at a concentration of 100 μmol/L. Effect of metabolites predicted to be lowered (A), increased (B) or unchanged (C) in Jurkat cells when compared with normal lymphoblasts, on the proliferation of Jurkat cells (2 biological replicates, each with 4 analytical replicates). MQ = menaquinone; HS = α-hydroxystearic acid; DE = dehydroepiandrosterone; SU = 3-sulfino-L-alanine; DM = 5,6-dimethylbenzimidazole; SE = seleno-L-methionine; RB = riboflavin; TN = tryptamine; HA = hydroxyacetone; BR = bilirubin; AT = androsterone; HV = homovanillic acid; VA = vanillylmandelic acid; AA = N-acetyl-L-aspartate; TA = taurocholic acid, CA = citric acid; PA = pantothenic acid; GA = β-D-galactose; FA = folic acid; CH = cholesterol. Error bars represent standard error of mean.

Based on simple criteria such as low molecular weight, commercial availability and affordability, we selected nine metabolites predicted to be lowered in Jurkat cells in order to test their effect on its proliferation (Table 1). For one of these nine metabolites (seleno-L-methionine) we carried out a validation by quantitative real-time PCR of the microarray data used by CoMet to make its prediction (see Additional File 3). We examined the effect on the growth of Jurkat cells of a 72 h treatment with riboflavin, tryptamine, 3-sulfino-L-alanine, menaquinone, dehydroepiandrosterone (the non-sulfated version of the predicted metabolite dehydroepiandrosterone sulfate), α-hydroxystearic acid (one of the possible compounds compatible with the predicted generic metabolite α-hydroxy fatty acid), hydroxyacetone, seleno-L-methionine and 5,6-dimethylbenzimidazole (the aglycone of the predicted metabolite α-ribazole) at a concentration of 100 μM (see Additional File 1). Figure 2A shows that all the tested metabolites with the exception of sulfino-L-alanine exhibited statistically significant antiproliferative activity on Jurkat cells (as evaluated by two-tailed t-tests at a critical alpha level of 0.05), with growth below 90% of the untreated control in all the cases. Although sulfino-L-alanine alone did not inhibit the growth of Jurkat cells, it significantly potentiated the inhibitory effect of seleno-L-methionine (43.1% to 30.3% growth compared to control). Similarly, a synergistic interaction between 5,6-dimethylbenzimidazole and seleno-L-methionine led to a supra-additive growth inhibitory activity. On the other hand, α-hydroxystearic acid and dehydroepiandrosterone show an additive effect, while α-hydroxystearic acid and seleno-L-methionine exhibited sub-additive or antagonistic inhibitory activity. Menaquinone showed the highest antiproliferative activity (11.3% growth compared to control), whereas the inhibitory activity of riboflavin, tryptamine and hydroxyacetone on Jurkat cells was more moderate (all above 70% growth compared to control). Thus, even with the small set tested, a remarkable richness of antiproliferative responses is seen.

### Most tested metabolites predicted to be augmented or unchanged in Jurkat cells are not antiproliferative

Although the fact that the nine tested metabolites predicted to be lowered in Jurkat cells exhibited antiproliferative activity strongly support our hypothesis, the possibility still exists that many endogenous metabolites at the concentration used in our test inhibit the growth of Jurkat cells, independent of the intracellular level status predicted by CoMet. Therefore, we tested metabolites whose intracellular levels in Jurkat cells were predicted to be increased (bilirubin, androsterone, homovanillic acid, vanillylmandelic acid, N-acetyl-L-aspartate and taurocholic acid) or unchanged (pantothenic acid, citric acid, folic acid, β-D-galactose, cholesterol) compared with lymphoblasts. We analyzed the effect on the growth of Jurkat cells of a 72 h treatment with each of the eleven human metabolites at a concentration of 100 μM (see Additional File 1). Figure 2B shows that only two of the six tested metabolites whose concentrations are predicted to be increased in Jurkat cells exhibit significant antiproliferative activity: bilirubin and androsterone (19.6% and 54.5% growth compared to control, respectively). The growth inhibition exerted by each of the remaining tested metabolites was less than 10% and statistically insignificant. Similarly, Figure 2C shows that all the tested metabolites whose intracellular levels in Jurkat cells and lymphoblasts we predict to be comparable, exhibit no significant effect on the growth of cells. Statistical significance was evaluated in all the cases according to two-tailed t-tests at a critical alpha level of 0.05.

### Predicted metabolites that are antiproliferative in Jurkat cells show lower or no activity in lymphoblasts

To validate the relevance of the identified antiproliferative metabolites as potential anticancer agents, we investigated the selectivity of their antiproliferative activity towards Jurkat cells with respect to human lymphoblast cells. We compared the effect of a 72 h treatment with menaquinone, dehydroepiandrosterone, seleno-L-methionine and 5,6-dimethylbenzimidazole at concentrations of 100 and 50 μM on the growth of Jurkat cells and human GM15851 lymphoblast cells cultured in the same conditions (see Additional File 1). Table 2 shows that menaquinone and dehydroepiandrosterone at concentrations of 100 or 50 μM exhibit significant antiproliferative activity on Jurkat cells but not on lymphoblast cells. Moreover, dehydroepiandrosterone at a concentration of 50 μM shows a statistically significant stimulation of the growth of lymphoblasts (130% growth compared to control), while at the same concentration in Jurkat cells the growth rate is reduced to 83.2%, thereby providing an interesting example of selective inhibition. Seleno-L-methionine inhibits the growth of Jurkat and lymphoblast cells at both tested concentrations; however, the effect on Jurkat cells was considerably more potent. While 5,6-dimethylbenzimidazole at a concentration of 100 μM exhibits significant antiproliferative activity on both tested cell lines, at a concentration of 50 μM it only inhibits Jurkat cells. Statistical significance was evaluated in all the cases according to two-tailed t-tests at a critical alpha level of 0.05.

**Table 2:** Differential effect of selected metabolites on the growth of Jurkat cells and normal lymphoblasts

		**Jurkat**			**Lymphoblasts**		
			
Metabolite	c [μM]^1^	Growth [%]	SEM [%]^2^	p-value	Growth [%]	SEM [%]^2^	p-value
Menaquinone	100	18.2	0.36	4.5 × 10^-7^	89.7	8.5	0.38
	50	40.3	1.15	2.0 × 10^-7^	109.9	5.8	0.23
Dehydroepiandrosterone	100	23.8	0.18	2.1 × 10^-6^	94.9	0.6	0.15
	50	83.2	0.23	2.0 × 10^-3^	130.8	6.9	0.020
Se-Seleno-L-methionine	100	41.0	0.67	1.0 × 10^-7^	79.3	5.8	0.028
	50	53.2	0.85	2.0 × 10^-3^	84.2	1.4	2.7 × 10^-4^
5,6-dimethylbenzimidazole	100	80.4	3.56	6.2 × 10^-3^	82.9	4.4	0.023
	50	87.8	1.41	0.021	97.4	1.9	0.037

^1 ^Metabolite concentration.

^2 ^Standard error of the mean.

^3 ^Statistical significance of the observed effect on cell growth, as evaluated by two-tailed t-tests.

## Discussion

### Novel antiproliferative compounds discovered by CoMet

At a concentration of 100 μmol/L, all nine metabolites predicted to be lowered in Jurkat cells selected for cell proliferation assays exhibited a statistically significant inhibition of Jurkat cell growth below 90% of the untreated control, whether alone, in most of the cases, or in combination, in the case of 3-sulfino-L-alanine (Figure 2A). On the other hand, 2/6 tested metabolites predicted to be increased in Jurkat cells unexpectedly exhibited antiproliferative activity (Figure 2B), while none of the five tested metabolites that we predicted to be unchanged inhibited Jurkat cell growth (Figure 2C). Thus, 18/20 assayed metabolites behave according to our working hypothesis. If we jointly consider the novel antiproliferative compounds presented in this work and those metabolites whose anticancer activity in Jurkat cells was previously known (discussed above), the fraction of anticancer metabolites among the compounds predicted to be lowered in Jurkat cells [(9+2)/104 = 0.106] is considerably higher than that corresponding to the rest of the compounds [(1+2+10+0)/(78+800) = 0.015]. A Fisher's exact test indicates that the positive association between lowered metabolite levels in Jurkat cells as predicted by CoMet and antiproliferative activity of the metabolite in that cell line is highly significant (two-tailed p-value = 8.7 × 10^-6^). These findings clearly support our hypothesis regarding the active role of endogenous metabolites in cancer, especially the concept that the metabolism of cancer cells may be regulated so that the intracellular concentration of certain antiproliferative metabolites is reduced, resulting in uninhibited cellular growth.

The growth inhibitory effects of some of the predicted compounds may seem relatively low and the tested concentrations of 50 and 100 μmol/L too high compared with most anticancer drugs of synthetic or natural origin. However, this range of concentrations is not unreasonable for metabolic compounds, since many metabolites can be found at similar levels in the cytosol and/or extracellular fluids [34]. Furthermore, when the effect of selected metabolites on growth inhibition was tested in Jurkat and lymphoblast cells cultured in identical conditions, a pattern of selectivity of the antiproliferative effect towards the cancer cell line became evident (Table 2). In an extreme case, dehydroepiandrosterone at a concentration of 50 μM inhibited the growth of Jurkat cells but stimulated the proliferation of lymphoblasts. Interestingly, several of our newly found antiproliferative metabolites exhibited synergistic interactions consistent with the Systems Biology approach of our method: the prediction was performed on the entire metabolome and not on individual metabolites or pathways. This raises the intriguing question of what the result would be if concentrations close to those observed in the normal cells could be achieved in the cancer cell for most metabolites, i.e. a reversion to a normal-like metabolic profile, at least for metabolites that inhibit cancer cell growth. If the antiproliferative activities we observed in a cancer cell line have therapeutic value, different combined strategies can be devised where sets of predicted metabolites are concurrently selected according to their association with the same or different metabolic pathways. For example, we can employ a strategy where multiple metabolites target a single pathway, or on the contrary, where each metabolite acts specifically on a different pathway. In addition, some active metabolites might serve as completely novel lead compounds for further drug design and development, with the advantage of reduced initial toxicity. While we have only performed cell proliferation assays, it is reasonable to speculate that some metabolites may also exhibit anti-metastatic (anti-invasive), anti-angiogenic, immunostimulant or other anticancer properties that would not be evident in an *in vitro *study of cell growth inhibition.

We did not investigate here the mode of action of the antiproliferative metabolites found by CoMet, and it is possible that some may exert their effect based on completely novel mechanisms, whose elucidation goes well beyond the immediate scope of this study. However, for most, we can suggest a possible mode of action based on their effect on other cancer cells or on the known properties of closely related molecules. For example, 5,6-dichlorobenzimidazole, a bioisosteric derivative of the active metabolite 5,6-dimethylbenzimidazole, induces differentiation of malignant erythroblasts by inhibiting RNA polymerase II [35]. The tested metabolite tryptamine is an effective inhibitor of HeLa cell growth via the competitive inhibition of tryptophanyl-tRNA synthetase, and consequent inhibition of protein biosynthesis [36]. 9-hydroxystearic acid, an isomer of the active metabolite α-hydroxystearic acid, arrests HT29 colon cancer cells in G0/G1 phase of the cell cycle via overexpression of p21 [37], induces differentiation of HT29 cells [38] by inhibition of histone deacetylase 1 [39] and interrupts the transduction of the mitogenic signal [40]. Menaquinone (vitamin K2), the most efficient compound among the tested metabolites, has been previously reported to induce G0/G1 arrest, differentiation and apoptosis in acute myelomonocytic leukemia HL-60 cells [41].

### Caveats and possible improvements of the approach

Besides the general problems that affect any microarray study [42,43], there are some factors not accounted in our method that can influence the actual intracellular levels of a metabolite. First, our qualitative treatment of metabolic flux is highly simplified; however, more quantitative approaches such as flux balance analysis require the knowledge of the regulatory effects of covalent modifications and the kinetic rate constants of the associated enzymes, information that is both incomplete and not accurate enough to generate large-scale models [44]. Second, the information available about both the subcellular location where the metabolic conversions take place and the transport of metabolites between different intra- or extracellular compartments is very limited. To partially address this issue, we are currently incorporating information about transporter genes into CoMet for qualitative metabolic flux predictions. Finally, a factor that could confound the hypothetical correlation between lowered metabolites in cancer and their potential as therapeutic agents is the existence of moonlighting activities, such as transcriptional regulation and apoptosis, exhibited by several metabolic enzymes [45]. Since the growth control functions of moonlighting enzymes do not involve catalysis, their regulated levels in cancer cells may not be correlated with the intracellular concentration of their metabolic substrates or products. Nevertheless, in spite of these in principle limitations, in practice, CoMet greatly succeeded in identifying antiproliferative metabolites. On the other hand, its ability to predict changes in intracellular metabolite concentrations still needs to be proven by experimentally determining the relative intracellular levels of a representative set of metabolites in normal and cancer cells, a task that we are planning to carry out in the near future.

## Conclusion

By applying CoMet, a Systems Biology based method for Computational Metabolomics, we have discovered eleven metabolites that either alone or in combination exhibit significant antiproliferative activity in Jurkat cells. The rationale behind our findings can be described by a simple premise: we posited that some metabolites that have lowered levels in a cancer cell as compared to normal cells might contribute to the progress of the disease. Our results strongly suggest that many other metabolites with important roles in cellular growth control may be waiting to be discovered, opening up the possibility of novel approaches against cancer. While traditionally cancer has been viewed as involving gene regulation, control and signaling processes that are disconnected from metabolism, in reality such processes are not disjoint; i.e., the cell is an integrated machine where such processes are likely to be highly coupled in many instances. CoMet adopts this viewpoint, and the resulting simple hypothesis that inspired its creation can greatly assist in the understanding of the contribution of metabolism to this complex disease.

## Methods

### Biological Databases

The main source of biological information is the Kyoto Encyclopedia of Genes and Genomes (KEGG) from July 5, 2007 [46]. We obtained the enzyme function annotation for human genes from the KEGG GENES database, the chemical information about human metabolites from the KEGG LIGAND database, and the metabolic pathway data from the KEGG PATHWAY database. Enzyme function annotations from KEGG were complemented by high confidence predictions made by EFICAz [47], our highly precise approach for enzyme function inference that significantly increases annotation coverage [48]. For the mapping between microarray probe identifiers and Entrez GeneID identifiers, the Affymetrix HG-U133 Plus 2.0 NetAffx Annotation file of May 31, 2007 was used [49].

### Gene expression status for enzyme-coding genes

The first step of the CoMet approach consists of the classification of each enzyme-coding human gene into four possible groups: G1) upregulated in cancer cells, G2) downregulated in cancer cells, G3) expressed in both, normal and cancer cells, at levels that are statistically indistinguishable, and G4) not expressed in both, normal and cancer cells. We used two types of data for the classification: the log base 2 signal intensities and the presence calls of the corresponding probe sets, as reported by the Affymetrix Microarray Suite Software 5.0 (MAS 5.0). First, an "off" status is assigned to each gene in each of the two studied conditions (normal and cancer) if the mean fraction of presence calls labeled as "marginal" or "absent" in the corresponding probe sets is at least 80%; otherwise, an "on" status is assigned. Then, each gene is classified into the G1, G2, G3 or G4 group, according to its on/off status in normal and cancer conditions and the following criterion for differential expression: the signal intensities in normal and cancer samples exhibit a statistically significant difference in at least 40% of the corresponding probe sets, as evaluated by an ANOVA two-tailed test with P < 0.005.

### Generation of genetic-metabolic matrix

The second step is the *in silico *estimation of the effect that the differentially expressed enzyme-encoding genes may exert on the intracellular levels of metabolites. First, all human metabolic pathways from the KEGG PATHWAY database, a compilation of maps representing the molecular interactions and reaction networks for different types of biological processes, were retrieved. For biological process labeled as Metabolism, there are eleven groups of pathways: 1) Carbohydrate Metabolism, 2) Energy Metabolism, 3) Lipid Metabolism, 4) Nucleotide Metabolism, 5) Amino Acid Metabolism, 6) Metabolism of Other Amino Acids, 7) Glycan Biosynthesis and Metabolism, 8) Biosynthesis of Polyketides and Nonribosomal Peptides, 9) Metabolism of Cofactors and Vitamins, 10) Biosynthesis of Secondary Metabolites, and 11) Xenobiotics Biodegradation and Metabolism. The pathway maps are available as graphical images and also as KEGG Markup Language (KGML) files that facilitate the parsing of relevant biological data. We extracted all the biochemical reactions from the KGML human metabolic pathway maps, including information about substrates, products, direction/reversibility and associated enzyme-coding genes. We then combined this information with gene expression data from normal and cancer cells to construct a genetic-metabolic matrix that links each of 1,477 metabolites with the specific human genes encoding for enzymes that consume and/or produce each metabolite, storing for each gene the differential expression status given by the four-group classification described in the previous section.

From the genetic-metabolic matrix, we excluded: i) 209 non-physiological metabolites, here defined as those that only participate in reactions that belong to the "Biosynthesis of Secondary Metabolites" and the "Xenobiotics Biodegradation and Metabolism" groups of metabolic pathways, e.g. ecgonine or parathion, ii) 90 metabolites that are considered ubiquitous and often carry out generic roles in many reactions [50], here defined as those that are involved as substrate or product in 20 or more reactions, e.g. H_2_O, ATP, NAD(^+^)(P) or O_2_, and iii) 289 metabolites that participate in reactions that are mainly catalyzed by orphan human enzymes. We define the number of reactions where a metabolite *m *acts as substrate or product in human metabolic pathways as Nr_*m*, human_, and in reference (non organism specific) metabolic pathways as Nr_*m*, ref_. If Nr_*m*, human_/Nr_*m*, ref _< 0.5, then the metabolite *m *belongs to the third exclusion category. The reactions absent in human pathways may be due to orphan enzymes, reactions that only occur in other organisms or reactions that may occur in humans but have not yet been detected. For example, the metabolite 1-alkyl-sn-glycero-3-phosphate is excluded because out of four enzymes that use it as substrate or product, two, EC 2.3.1.105 and EC 1.1.1.101, are orphans in human, and one, EC 2.7.1.93, has only been found in rabbit [51]. The total number of metabolites remaining in the genetic-metabolic matrix after the three types of exclusion is 982 (see Additional File 2).

### Scanning of genetic-metabolic matrix

In the last step, we apply a simple set of rules to scan the genetic-metabolic matrix for metabolites whose intracellular levels in cancer cells are likely to differ from those in normal cells. The rules are based on the supposition that lower levels of enzymes catalyzing the production of a metabolite (or higher levels of enzymes catalyzing the consumption of it) imply decreased level of such metabolite, and vice versa (see Figure 1). In our methodology, a given metabolite is predicted to have decreased levels in cancer cells when: 1) both of the following apply: 1.1) there is no gene encoding for an enzyme able to catalyze the production of the metabolite whose differential expression status is G1 (upregulated in cancer cells) or G3 (significantly expressed at similar levels in normal and cancer cells) and 1.2) there is no gene encoding for an enzyme able to catalyze the consumption of the metabolite whose differential expression status is G2 (downregulated in cancer cells), and 2) either or both of the following applies: 2.1) there is at least one gene encoding for an enzyme able to catalyze the production of the metabolite whose differential expression status is G2 (downregulated in cancer cells) and 2.2) there is at least one gene encoding for an enzyme able to catalyze the consumption of the metabolite whose differential expression status is G1 (upregulated in cancer cells). Similarly, a metabolite is predicted to have increased levels in cancer cells when: 1) both of the following applies: 1.1) there is no gene encoding for an enzyme able to catalyze the consumption of the metabolite whose differential expression status is G1 or G3 and 1.2) there is no gene encoding for an enzyme able to catalyze the production of the metabolite whose differential expression status is G2, and 2) either or both of the following applies: 2.1) there is at least one gene encoding for an enzyme able to catalyze the consumption of the metabolite whose differential expression status is G2 and 2.2) there is at least one gene encoding for an enzyme able to catalyze the production of the metabolite whose differential expression status is G1. Thus, the methodology attempts to consider, as much as is practical, the entire proteome complement of enzymes that produce and consume the metabolite. That is, our approach is Systems Biology based.

## Competing interests

We are currently applying for a patent related to the content of this work.

## Authors' contributions

AKA and JS jointly formulated the idea of CoMet and the hypothesis that downregulated metabolites should be appropriate anticancer targets, AKA developed CoMet, participated in the design of the study and drafted the manuscript, RM carried out cell cultures, antiproliferative activity and qRT-PCR experiments, participated in the design of the study and helped to draft the manuscript, RM and AKA interpreted the experimental results, NJB carried out the microarray experiments, YH participated in the development of CoMet, JFM and JS conceived of the study, participated in its design and coordination and helped to draft the manuscript. All authors have read and approved the final manuscript.

## Supplementary Material

Additional File 1 Supplementary Methods. Word document describing the cell cultures, RNA extraction, amplification and microarray data processing, cell proliferation assays and verification of selective antiproliferative effect of metabolites on Jurkat vs. lymphoblast cells. Click here for file

Additional File 2 Metabolites present in the genetic-metabolic matrix. Excel table listing KEGG Ligand Identifier, common names and hyperlink to KEGG database for the 982 metabolites present in the genetic-metabolic matrix, as well as the 104 (78) metabolites whose concentration is predicted by CoMet to be lowered (increased) in Jurkat cells compared to normal lymphoblasts. Click here for file

Additional File 3 Validation of microarray gene expression data by qRT-PCR. Word document describing the validation by quantitative real-time PCR (qRT-PCR) of the microarray gene expression data corresponding to the genes used by CoMet to predict a decreased level of seleno-L-methionine in Jurkat cells. Click here for file
