# Supplementary material for: Identification of metabolites with anticancer properties by computational metabolomics
Source: Mol Cancer. 2008 Jun 17;7:57. doi: 10.1186/1476-4598-7-57 (PMC2453147; doi:10.1186/1476-4598-7-57)
Supplement: Additional File 1 — Supplementary Methods. Word document describing the cell cultures, RNA extraction, amplification and microarray data processing, cell proliferation assays and verification of selective antiproliferative effect of metabolites on Jurkat vs. lymphoblast cells. [file 1476-4598-7-57-S1.doc]

# Supplementary Methods

## Cell cultures

Human T-acute lymphoblastic leukemia Jurkat procured from ATCC were grown at RPMI-1640 medium (Mediatech) supplemented with 10% FBS (Gibco), 2 mmol/L L-glutamine (Mediatech), 100 IU/mL penicillin, 100 μg/mL streptomycin, and 0.25 μg/mL amphotericin B (all from Mediatech) at 37°C in the atmosphere of 5% CO_2_, 95% air and 80% relative humidity. Approximately 10^6^ exponentially growing cells from two biological replicates were used for the isolation of total cellular RNA.

## RNA extraction, amplification and microarray data processing

Total RNA was extracted from Jurkat cells using Trizol (Invitrogen) and processed using the RiboAmp OA or HS kit (Arcturus) in conjunction with the IVT Labeling Kit from Affymetrix, to produce an amplified, biotin-labeled mRNA suitable for hybridizing to GeneChip Probe Arrays (Affymetrix). Labeled mRNA was hybridized to GeneChip Human Genome U133 Plus 2.0 Arrays in the GeneChip Hybridization oven 640, further processed with the GeneChip Fluidics Station 450 and scanned with the GeneChip Scanner. Affymetrix CEL files were processed using the Affymetrix Expression Console (EC) Software Version 1.1. Files were processed using the default MAS5 3’ expression workflow, which includes scaling all probes to a target intensity (TGT) of 500. Spiked in report controls used were AFFX-BioB, AFFX-BioC, AFFX-BioDn, and AFFX-CreX. Affymetrix CEL files for two Jurkat cell samples were submitted to Gene Expression Omnibus (samples GSM243727 and GSM243728, grouped into Series GSE9648) [1]. A link has been created to allow anonymous review of GSE9648 before its public release at the time of publication [2]. Affymetrix CEL files for three normal lymphoblast samples [3] used as a normal reference to compare Jurkat cells expression data were directly retrieved from the Gene Expression Omnibus (samples GSM113678, GSM113802 and GSM113803 of untreated GM15851 cells from Series GSE5040).

## Cell proliferation assays

Growth inhibition of Jurkat cells was evaluated by a resazurin-based [4] in vitro toxicology assay kit (Sigma-Aldrich). Stock solutions (40 mmol/L) of the following metabolites were stored frozen at -80°C prior to its use: dehydroepiandrosterone (dehydroisoandrosterone, Acros Organics), 5,6-dimethylbenzimidazole (Sigma-Aldrich), hydroxyacetone (Sigma-Aldrich), menaquinone (Supelco), riboflavin (Sigma-Aldrich), tryptamine (Sigma-Aldrich), α-hydroxystearic acid (Sigma-Aldrich), bilirubin (Sigma-Aldrich, mixed isomers), androsterone (Sigma-Aldrich), homovanillic acid (4-hydroxy-3-methoxy-benzeneacetic acid, Sigma-Aldrich) and vanillylmandelic acid (D,L-4-hydroxy-3-methoxymandelic acid, Sigma-Aldrich) solubilized in DMSO (Sigma-Aldrich), 3-sulfino-L-alanine (L-cysteinesulfinic acid, Sigma-Aldrich), seleno-L-methionine (Sigma-Aldrich), N-acetyl-L-aspartate (Fluca), taurocholic acid (taurocholic acid sodium salt hydrate, Sigma-Aldrich), citric acid (citric acid trisodium salt dihydrate, Sigma-Aldrich), pantothenic acid (D-pantothenic acid, Sigma-Aldrich), β-D-galactose (D-(+)-galactose, Sigma-Aldrich) and folic acid (Sigma-Aldrich) solubilized in sterile deionized water and cholesterol (Sigma-Aldrich) solubilized in absolute ethyl alcohol. Aliquots of 100 μL of cells in phenol red-free RPMI 1640 medium (Sigma) supplemented with 5% FBS, 2 mmol/L L-glutamine, 100 IU/mL penicillin, 100 μg/mL streptomycin, and 0.25 μg/mL amphotericin B were inoculated into 96-well black-walled plates at a density of 1.5x10^5^ cells/mL and incubated for 24 h at 37°C in 5% CO_2_, 95% air and 80% relative humidity prior to the addition of the metabolites to be tested. Stock solutions of metabolites were diluted 200 times with complete growth medium and added to the appropriate microtiter wells in 4 replicates per metabolite, while 100 μL of complete medium was added to the control and blank cells. Following metabolite addition, the plates were incubated for an additional 72 h, after which 20μL of TOX-8 reagent for was added to metabolite treatment, control and blank wells and incubation continued for additional 3 h. The increase in fluorescence was measured in a microplate fluorimeter at 590 nm using an excitation wavelength of 560 nm. The emission of control wells, after the subtraction of a blank, was taken as 100% and the results for metabolite treatments were expressed as percentage of the control. Two biological replicates for each cell line were used for cell proliferation assays. Positive results were additionally verified by counting of viable cells using Vi-CELL XR cell counter (Beckman Coulter) and trypan blue dye exclusion method for Jurkat cells.

## Verification of selective cytostatic/cytotoxic effect of metabolites on Jurkat vs. lymphoblast cells

Non-leukemic human lymphoblasts (Coriell, Camden, NJ, catalogue # GM 15851) and Jurkat cells were grown on RPMI-1640 with L-glutamine medium (Cellgro) supplemented with 15% FBS and Antibiotic-Antimycotic solution (Cellgro) at 37°C in the atmosphere of 5% CO2, 95% air and 80% relative humidity. Antiproliferative effects of menaquinone, seleno-L-methionine, dehydroepiandrosterone and 5,6-dimethylbenzimidazole at final concentrations of 100 and 50 µM were evaluated as described in the **Cell proliferation assays** section.

# References
