# Supplementary material for: Identification of metabolites with anticancer properties by computational metabolomics
Source: Mol Cancer. 2008 Jun 17;7:57. doi: 10.1186/1476-4598-7-57 (PMC2453147; doi:10.1186/1476-4598-7-57)
Supplement: Additional File 3 — Validation of microarray gene expression data by qRT-PCR. Word document describing the validation by quantitative real-time PCR (qRT-PCR) of the microarray gene expression data corresponding to the genes used by CoMet to predict a decreased level of seleno-L-methionine in Jurkat cells. [file 1476-4598-7-57-S3.doc]

# Validation of microarray gene expression analysis by qRT-PCR

## Background

CoMet’s prediction of decreased levels of seleno-L-methionine in Jurkat cells compared to human lymphoblasts is based on the comparative analysis of microarray expression data for the genes MAT2A (the alpha subunit of methionine adenosyltransferase 2), MAT2B (the beta subunit of methionine adenosyltransferase 2), MARS (methionyl tRNA synthetase) and MARS2 (methionyl tRNA synthetase 2) in these cell lines. Under physiological conditions, the enzymes encoded by these four genes catalyze reactions that consume seleno-L-methionine. According to CoMet’s analysis of the microarray data, three of these genes (MAT2B, MARS and MARS2) are expressed at similar levels in normal and cancer cells, while one of them (MAT2A) is upregulated in cancer cells. To validate the changes in gene expression inferred from microarray data, we determined the transcript levels of the four genes in Jurkat cells and human lymphoblasts by quantitative real-time PCR (qRT-PCR).

## Methods

Human lymphoblasts (Coriell, Camden, NJ, catalogue # GM 15851) and human T-acute lymphoblastic leukemia Jurkat cells (procured from ATCC) were grown on RPMI-1640 medium with L-glutamine (Cellgro) supplemented with 15% FBS (Gibco) and Antibiotic-Antimycotic solution (Cellgro) at 37°C in the atmosphere of 5% CO_2_, 95% air and 80% relative humidity. Cells in the exponential phase of growth were collected and total cellular RNA was isolated using the RNeasy Mini Kit (Qiagen). We followed the manufacturer’s protocols for purification of total RNA from animal cells and RNA cleanup. Quality control of isolated RNA was performed using the Agilent 2100 Bioanalyzer (Agilent Technologies). First-strand cDNA was synthesized from about 2 µg of total lymphoblast and Jurkat RNAs using SuperScript III First-Strand Synthesis System for RT-PCR in accordance with protocol for oligo(dT)20 primer (Invitrogen). Expression analysis of MAT2A, MAT2B, MARS2 and MARS genes in Jurkat and lymphoblast cells relative to GAPDH endogenous control was performed by qPCR in DNA Engine Opticon 2 Continuous Fluorescence Detection System (Bio-Rad), using TaqMan Universal PCR Master Kit and TaqMan Gene Expression Assays Hs00428515_g1, Hs00203231_m1, Hs00536599_s1, Hs00192202_m1 and Hs99999905_m1, respectively (all from Applied Biosystems). PCR reactions for each gene and cell line were performed in quadruplicates with no-reverse transcription control for genomic DNA amplification. The relative expression of each gene in Jurkat compared to lymphoblast cells was calculated from the measured threshold cycle values by means of the relative expression software tool REST basis v2 (downloaded from www.Gene-Quantification.info).

## Results and Discussion

According to the qRT-PCR analysis, the only gene that exhibits a significant difference in relative expression is MAT2A (p-value=0.045), with transcript levels in Jurkat cells that are 2.87 higher than those in human lymphoblasts. The relative levels of MAT2B, MARS and MARS2 transcripts in Jurkat vs. lymphoblasts are 1.19 (p-value=0.28), 1.20 (p-value=0.67) and 1.23 (p-value=0.38), respectively. In all the cases, the statistical significance was evaluated by a two-tailed Pair Wise Fixed Reallocation Randomization Test [1] at a critical alpha value of 0.05. Thus, the qRT-PCR analysis fully agrees with the changes in gene expression calculated from the microarray data.

# References

1. Pfaffl MW, Horgan GW, Dempfle L**: Relative expression software tool (REST) for group-wise comparison and statistical analysis of relative expression results in real-time PC**R*. Nucleic Acids Re*s 2002**, 3**0:e36.
